# Retraction: Qian, W., et al. Sanguinarine Inhibits Mono- and Dual-Species Biofilm Formation by *Candida albicans* and *Staphylococcus aureus* and Induces Mature Hypha Transition of *C. albicans*. *Pharmacueticals* 2020, *13*, 13

**DOI:** 10.3390/ph14030193

**Published:** 2021-02-26

**Authors:** Pharmaceuticals Editorial Office

**Affiliations:** MDPI, St. Alban-Anlage 66, 4052 Basel, Switzerland; pharmaceuticals@mdpi.com

The journal retracts the article [1] cited above.

Following publication, concerns were brought to the attention of the publisher regarding the improper use of images. The FESEM image of dual-culture (SA+CA) biofilms under the 1/2 MIC in Figure 2 is a duplication of the image under 32 MIC in Figure 7. The second duplication concerns two images published in two papers: Figure 2A in paper [1] is duplicated from Figure 2A in the published paper [2].

Adhering to our complaints procedure, an investigation was conducted that confirmed the extent of the issues, and the article is therefore retracted.

This retraction was approved by the Editor in Chief of the journal.

The authors agreed to this retraction.
